# Investigation of ENO2 as a promising novel marker for the progression of colorectal cancer with microsatellite instability-high

**DOI:** 10.1186/s12885-024-12332-4

**Published:** 2024-05-09

**Authors:** Junwen Cai, Yuting Yang, Leilei Zhang, Yangyang Fang, Yanjun Zhang, Mingyue Tan, Juan Zhang, Chen Tang, Haitao Ren, Lanni Wang, Guangxin Xiang, Feng Xu, Linhua Lan, Liyi Li, Xiaoqun Zheng

**Affiliations:** 1 Department of Clinical Laboratory, the Second Affiliated Hospital and Yuying Children’s Hospital of Wenzhou Medical University, Wenzhou, 325000 China; 2 School of Laboratory Medicine and Life Sciences, Wenzhou Medical University, Wenzhou, 325000 China; 3 Key Laboratory of Diagnosis and Treatment of Severe Hepato-Pancreatic Diseases of Zhejiang Province, The First Affiliated Hospital of Wenzhou Medical University, Wenzhou, 325000 China; 4 General Surgery Department, Second Affiliated Hospital of Wenzhou Medical University, Wenzhou, 325000 China; 5 The Key Laboratory of Laboratory Medicine, Ministry of Education, Wenzhou, 325035 China

**Keywords:** ENO2, Microsatellite instability, Colorectal cancer, Biomarker

## Abstract

**Background:**

Microsatellite instability-high (MSI-H) has emerged as a significant biological characteristic of colorectal cancer (CRC). Studies reported that MSI-H CRC generally had a better prognosis than microsatellite stable (MSS)/microsatellite instability-low (MSI-L) CRC, but some MSI-H CRC patients exhibited distinctive molecular characteristics and experienced a less favorable prognosis. In this study, our objective was to explore the metabolic transcript-related subtypes of MSI-H CRC and identify a biomarker for predicting survival outcomes.

**Methods:**

Single-cell RNA sequencing (scRNA-seq) data of MSI-H CRC patients were obtained from the Gene Expression Omnibus (GEO) database. By utilizing the copy number variation (CNV) score, a malignant cell subpopulation was identified at the single-cell level. The metabolic landscape of various cell types was examined using metabolic pathway gene sets. Subsequently, functional experiments were conducted to investigate the biological significance of the hub gene in MSI-H CRC. Finally, the predictive potential of the hub gene was assessed using a nomogram.

**Results:**

This study revealed a malignant tumor cell subpopulation from the single-cell RNA sequencing (scRNA-seq) data. MSI-H CRC was clustered into two subtypes based on the expression profiles of metabolism-related genes, and *ENO2* was identified as a hub gene. Functional experiments with *ENO2* knockdown and overexpression demonstrated its role in promoting CRC cell migration, invasion, glycolysis, and epithelial-mesenchymal transition (EMT) in vitro. High expression of ENO2 in MSI-H CRC patients was associated with worse clinical outcomes, including increased tumor invasion depth (*p* = 0.007) and greater likelihood of perineural invasion (*p* = 0.015). Furthermore, the nomogram and calibration curves based on *ENO2* showed potential prognosis predictive performance.

**Conclusion:**

Our findings suggest that ENO2 serves as a novel prognostic biomarker and is associated with the progression of MSI-H CRC.

**Supplementary Information:**

The online version contains supplementary material available at 10.1186/s12885-024-12332-4.

## Background

Colorectal cancer (CRC) is a prevalent category of gastrointestinal malignancies with high incidence and significant heterogeneity [1]. Microsatellite typing of CRC plays a crucial role in the treatment and prognosis determination. Microsatellite instability-high (MSI-H) in CRC patients is associated with a favorable prognosis [2]. However, some MSI-H CRC patients exhibit distinctive molecular characteristics and experience a less favorable prognosis [3, 4]. This result highlights the considerable heterogeneity within MSI-H CRC and underscores the need for more precise subtyping and novel biomarker to guide treatment strategies.

Metabolic reprogramming, which includes phenomena like aerobic glycolysis, disruptions in lipid synthesis and breakdown, and heightened amino acid metabolism, has been implicated in the initiation and progression of cancer within the tumor microenvironment [5]. These abnormal metabolic adaptations provide tumor cells with an ample supply of energy, nutrients, and redox balance, which in turn facilitates their malignant growth and facilitates metastasis [6]. In recent years, various studies have attempted to classify tumors into different subtypes based on distinct patterns of gene expression related to metabolism. Prognostic indicators rooted in metabolic gene sets have been proposed for malignancies such as head and neck cancer [7], as well as lung adenocarcinoma [8]. However, the characterization of metabolic-related gene subtypes in MSI-H CRC remains limited.

In this study, using the data collected from the GSE59857 dataset, we conducted a clustering analysis for the expression profiles of genes related to metabolism. Then, we elucidated the underlying pathway enrichment between subtypes and compared the prognosis. Furthermore, *ENO2* was identified as a marker gene for the C2 subtype. To explore the role of *ENO2* in the progression of MSI-H CRC, we assessed the effect of *ENO2* on wound healing, transwell migration, invasion and glycolysis in cell cultures and scrutinized potential signaling pathways that might be related to these observed effects. Additionally, we analyzed the ENO2 expression of CRC tissues from patients, to verify its predictive values for CRC prognosis.

## Methods

### Data collection

MSI-H CRC scRNA-seq datasets were accessed from GSE178341(*n* = 34) via the Gene Expression Omnibus (GEO) database (https://www.ncbi.nlm.nih.gov/geo/). Bulk transcriptome data of MSI-H CRC cell line was downloaded from GSE59857(*n* = 60). The RNA-seq data and corresponding clinical information of MSI-H CRC were obtained from The Cancer Genome Atlas (TCGA) database (https://portal.gdc.cancer.gov/).

### Single-cell RNA-seq analysis

The Seurat R package (version 4.3.0.1) was used to perform a standardized analysis (https://github.com/satijalab/seurat) [9]. Genes that were detected in less than 10 cells as well as cells with less than 200 detected gene numbers were ruled out, and the proportion of mitochondria was limited to less than 10%. Then, the LogNormalize method was applied for data normalization. Uniform manifold approximation and projection (UMAP) was utilized after principal component analysis (PCA) for unsupervised clustering and unbiasedly visualizing cell populations on a two-dimensional map [10].

The CNV score was calculated by the inferCNV R package (Version 1.6.0, https://github.com/broadinstitute/infercnv) to evaluate chromosomal variation [11]. To quantify the metabolic scores of variable cell subpopulations, we referred to a publicly available method [12]. All related code was available on GitHub at https://gitmarker.com/LocasaleLab/Single-Cell-MetabolicLandscape. We applied Pearson correlation analysis to identify cell subpopulations from single-cell data that were most highly associated with the metabolic molecular subtypes. Subsequently, the “FindAllMarkers” function was utilized to identify marker genes of each cluster with the filter value of absolute log2 fold change (|log2FC|) ≥ 1 and the minimum cell population fraction in either of the two populations was 0.1.

### Bulk transcriptome analysis

For cell line data, we extracted metabolic gene expression profiles to construct molecular subtypes and UMAP was used for dimensionality reduction clustering. The limma R package(version 3.56.2) was utilized to calculate the differentially expressed genes (DEGs) among subclasses with the filter value of adjusted *p* value < 0.05 and |log2FC|≥ 1 [13]. Gene set enrichment analysis (GSEA)was performed with The Molecular Signatures Database (MSigDB) (h.all.v2023.1.Hs.symbols, c2.cp. Reactome.v2023.1.Hs.symbols) via fgsea R package (version 1.12.0) [14].

For TCGA data, all patients with MSI-H CRC were divided into two groups based on a gene centroid classifier to perform survival analysis. Differential gene analysis was the same as cell line data. Gene set enrichment analysis (GSEA) was performed with the Kyoto Encyclopedia of Genes and Genomes(KEGG)database and MSigDB via clusterProfiler(version 4.8.2) [15].

### Generation of the classifier

To identify subtypes in novel cohorts using a small list of genes, we developed a gene expression-based classifier. Differentially expressed genes were further trained by prediction analysis for microarrays (PAM) to build a classifier using the R package ‘‘pamr’(version 1.56.1) [16]. The transcriptome data was normalized prior to classification. Subclass Mapping (SubMap) analysis (GenePattern module 15 SubMAP, https://cloud.genepattern.org) was used to identify common subgroups between independent cohorts despite their technical differences [17].

### Patient specimens

In this study, a total of 49 paraffin sections from patients with MSI-H CRC and adjacent non-tumorous tissues were obtained from the Second Affiliated Hospital of Wenzhou Medical University between January 2019 and June 2022. These sections were used to measure protein levels. Prior to participation, all patients provided informed consent, and the study was approved by the Ethics Committee of the Second Affiliated Hospital of Wenzhou Medical University.

### Cell culture and transfection

HEK293 cells, FHC cells, MSS cell lines (SW620, HT-29) and MSI-H CRC cell lines (LS-174 T, GP2D, RKO, LOVO) were obtained from the Cell Bank of the Chinese Academy of Sciences (Shanghai, China). HEK293, SW620, HT-29, LS-174 T, and GP2D cells were cultured in Dulbecco's Modified Eagle Medium, while FHC and RKO cells were cultured in Roswell Park Memorial Institute-1640 medium, and LOVO cells were cultured in Ham's F-12 K medium. All culture media were supplemented with 10% fetal bovine serum (10099141, Gibco™) and antibiotics (100 U/ml penicillin and 100 μg/ml streptomycin, C0222, Beyotime) and maintained at 37 °C with 5% CO_2_.

For transfection, shRNA (PPL, China) and lipofectamine_3000 transfection reagent (Invitrogen, USA) were used following the manufacturer's protocols.

### Wound healing assay

The transfected cells were seeded into 6-well plates at a concentration of 3–7 × 10^5^ cells/ml. When the cells reached approximately 90% confluency, a straight wound was created on the confluent monolayer using a 10 μL pipette tip. The cells were then cultured in serum-free medium for 48 h. After that, the wounded monolayer was washed with phosphate-buffered saline (PBS) and photographed using an inverted microscope.

### Transwell migration and invasion assays

Migration and invasion assays were conducted using 24-well plates with inserts (3540, Corning) with or without Matrigel. CRC cells (2 × 10^4^ cells/well) were added to the upper chambers in serum-free media. Simultaneously, RPMI 1640 containing 10% FBS was added to the lower chambers. Following incubation at 37 °C in 5% CO_2_ for 24 h (migration assay) or 48 h (invasion assay), the upper chamber was wiped with a cotton swab, and the lower chamber was fixed with 4% paraformaldehyde. Subsequently, it was stained with 0.1% crystal violet and washed three times with water.

### Western blot (WB) analysis

The collected protein lysates were separated using sodium dodecyl-sulfate–polyacrylamide (10%–15%) gels with electrophoresis. The wet transfer method was applied for transferring proteins to the polyvinylidene difluoride (PVDF) membrane. The immobilized proteins on the membrane were then blocked with 5% milk solution prepared in 0.1% Tween-20 in Tris-buffered saline (TBS-T) for 1 h. The membranes were cut and incubated with the corresponding antibodies (1 h at room temperature or overnight at 4℃). The primary antibodies were then washed with TBS-T solution for half an hour and replaced with a secondary antibody conjugated with HRP, which was also washed subsequently. Immunoreactive proteins were detected by ECL reagent according to the manufacturer's protocol (1705061, Bio-Rad).

### Immunohistochemistry (IHC) and immunofluorescence (IF) assay

IHC staining of paraffin-embedded tissues with antibody against ENO2 (1:200, 66150–1-Ig, Proteintech) was performed following the standard procedures as previously described [18].

Cells were fixed with 4% paraformaldehyde, permeabilized with 0.2% Triton X-100, and stained according to standard procedures. Cells were incubated with anti-Slug primary antibody (1:100,12129–1-AP, Proteintech) overnight at 4 °C and then washed with PBS. DAPI reagent was used to stain cell nuclei. Finally, the cells were observed under an inverted fluorescent microscope.

### Glycolysis stress test

A Seahorse XF96 Extracellular Flux Analyzer (Agilent Technologies, USA) was used for extracellular acidification rate (ECAR) measurements. 2 × 10^4^ cells add to machine-specific plates. Drug doses were used to treat cells for 6 h. Following probe calibration, ECAR was measured via sequentially injecting glucose (10 mM), oligomycin (1 µM), or 2-DG (100 mM).

### Statistical analysis

Survival analyses were conducted using the Kaplan–Meier method and compared using the log-rank test. The chi-squared test was used to analyze the correlations between ENO2 expression and clinicopathological factors. For comparisons between two groups with normally distributed variables, the unpaired Student's t-test was employed, while one-way analysis of variance was used for comparisons among three groups. Statistical analysis was performed using R (version 4.3.1). Experimental data in the article were analyzed using GraphPad Prism 9.0 software. A *p*-value < 0.05 was considered statistically significant.

## Results

### A highly metabolically active subset of malignant cells unveiled through single-cell RNA sequencing

**Fig. 1:**
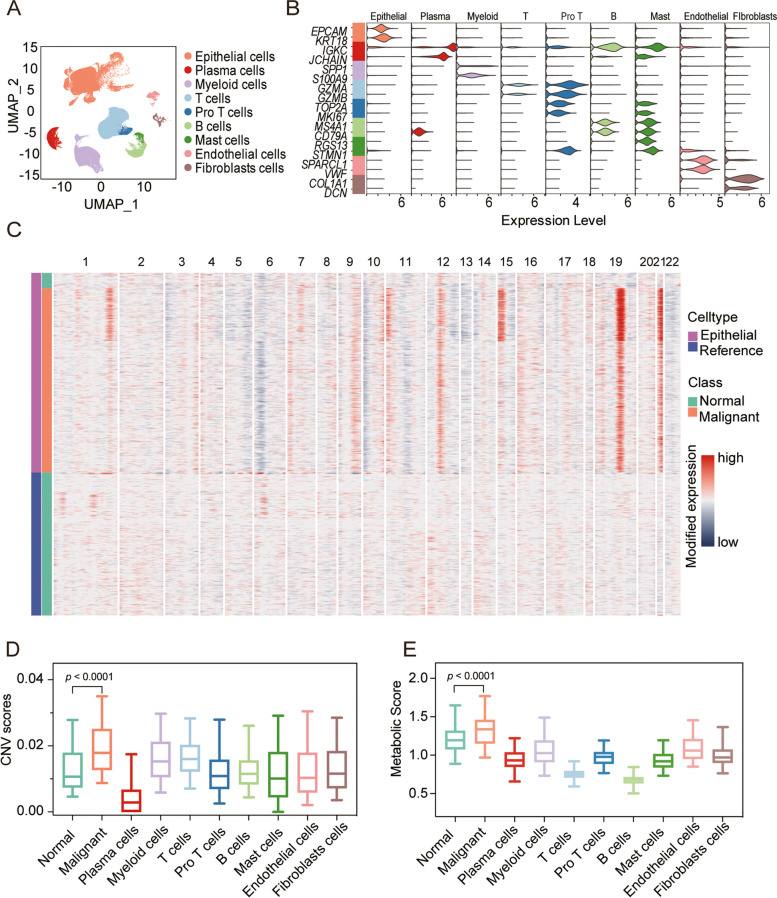
Identifying a malignant cell subpopulation in colorectal cancer with microsatellite instability-high (MSI-H CRC). **A** UMAP dimensional reduction showing major cell types in 34 patients with MSI-H CRC. **B** Violin plots for marker genes of different cell subsets. **C** Copy number variation (CNV) heatmap of epithelial cells. **D** Violin plots for copy number variation (CNV) scores of each cell type. Wilcoxon rank test, *p* < 0.0001. **E** Violin plots for metabolic scores of each cell type. Wilcoxon rank test, *p* < 0.0001

ScRNA-seq data were obtained from 34 patients with MSI-H CRC, and 9 different clusters including T cells, pro T cells, B cells, myeloid cells, mast cells, plasma cells, fibroblast cells, endothelial cells, and epithelial cells were finally identified by standardizing the single-cell processing steps. The marker genes for each cell cluster were consistent with known cellular markers (Fig. 1A and B). To further identify malignant cells, the variable copy number (CNV) score was calculated via the InferCNV package, and different patterns of chromosomal copy number variation in the epithelial cells were revealed (Fig. 1C). A subset with high CNV scores was identified as malignant tumor cells (Fig. 1D). Taking a publicly available method as reference, the metabolic scores of different cell types were quantified. Showing that malignant cells had a more active metabolic pathway than other cells (Fig. 1E). This result suggested that the metabolic activity was correlated with the malignant progression of tumor cells.

### MSI-H CRC classified into two distinct subtypes with *ENO2* identified as a central hub gene

**Fig. 2:**
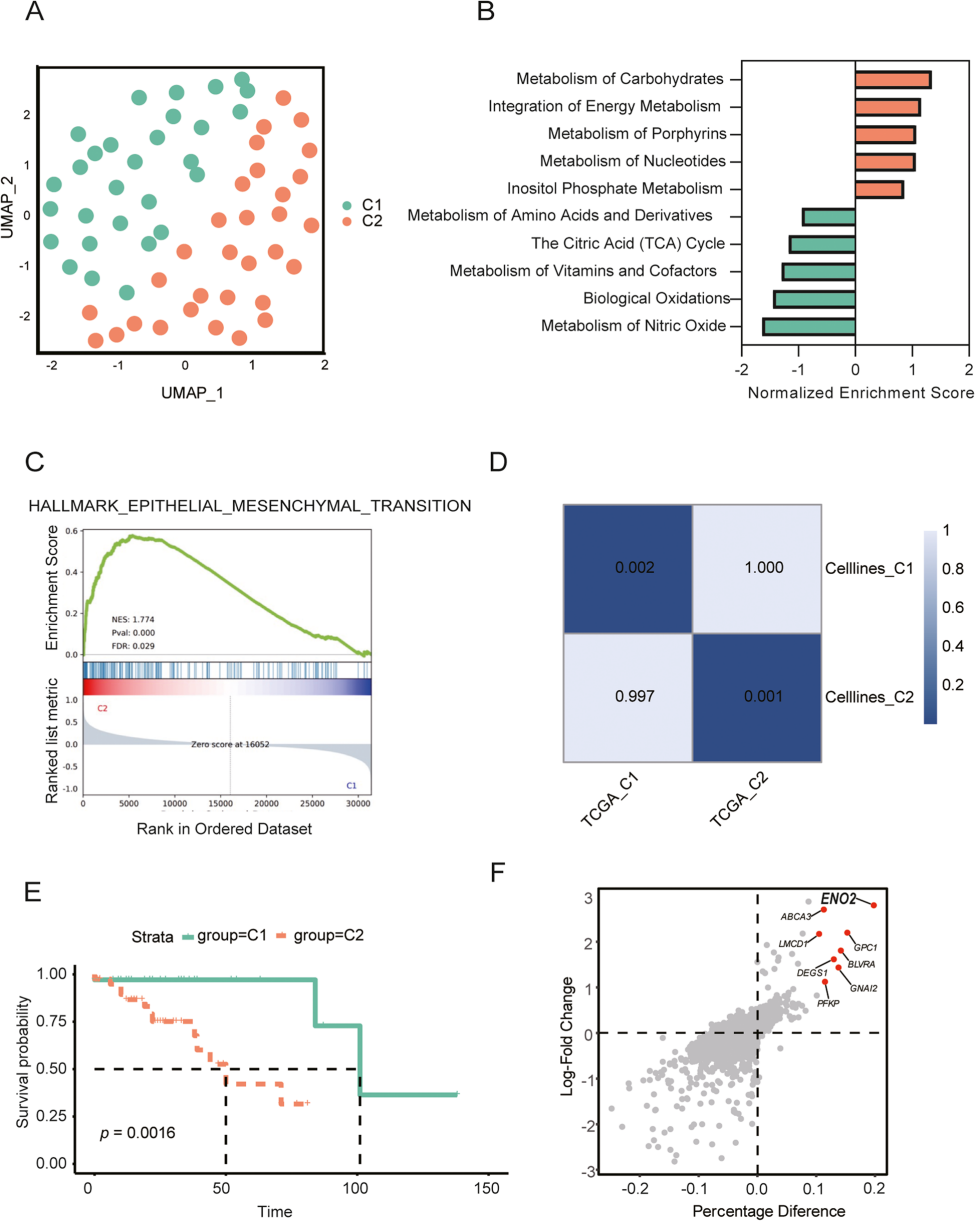
Identification biomarkers of MSI-H CRC typing and prognosis. **A** UMAP dimensional reduction showing 60 cell lines of MSI-H CRC. **B** Metabolic pathway enrichment fraction histogram. **C** The EMT pathway in MSI-H CRC. **D** SubMAP heatmap of corrected *p*-value. **E** Kaplan-Meier analysis in term of OS in TCGA cohort. **F** Scatter plot of differentially expressed genes

Transcriptomic data from cell lines were meticulously gathered to unveil heterogeneous metabolic molecular clusters within the GSE59857 dataset. For this analysis, we carefully selected 2,752 metabolism-related genes representing various metabolic processes, which were served as the basis for the subsequent dimensionality reduction clustering [19]. In this cohort, 29 cell lines were categorized into cluster 1, while 31 cell lines were allocated to cluster 2, maintaining an approximate 1:1 ratio (Fig. 2A).

We conducted GSEA to gain further insights into the metabolic characteristics of these subgroups. Our findings demonstrated that cluster 1 (C1) exhibited enriched pathways related to the amino acid and derivatives metabolism, the tricarboxylic acid cyclecycle (TCA), and the biological oxidations, whereas cluster 2 (C2) demonstrated enrichment in metabolic pathways, including carbohydrate metabolism and the integration of energy metabolism, among others (Fig. 2B). Notably, the EMT pathway, which played a pivotal role in CRC progression, was upregulated in C2, suggesting that this subtype may be highly malignant (Fig. 2C) [20, 21]. To identify subtypes in novel datasets, a gene classifier based on centroid was developed with reference to an article [16].We constructed a PAM classifier based on the differential genes of cell lines and apply it to the typing of TCGA cohort. Subclass Mapping (SubMap) analysis confirmed that each subtype was associated with similar underlying transcriptional traits in the MSI-H CRC of TCGA cohort (Fig. 2D).Survival analysis showed that patients with C2 subtype had shorter overall survival and poorer prognosis than C1 subtype(Fig. 2E). Subsequently, we subdivided malignant tumor cells into two distinct categories at the single-cell level (Fig. S1A).

In our pursuit of uncovering the underlying gene expression distinctions between these subtypes, we conducted a comprehensive analysis of Differentially Expressed Genes (DEGs). This analysis between cluster 1 and cluster 2 unveiled a greater number of marker genes that were notably expressed in cluster 2, including *ENO2*, *ABCA3*, and *GPC1* (Fig. 2F). *ENO2* was highly expressed in both the C2 subtype of the cell line and the C2 subtype of TCGA, suggesting that *ENO2* is a marker gene for the C2 subtype (Fig. S1B and C). Thus, *ENO2* is identified as a central hub gene. We explored the prognostic significance of ENO2, the combination of ENO2 with MSI seemed to have a better prognostic capability for CRC patients, and patients with high expression of ENO2 and MSI-L/MSS tended to have the worst prognosis(Fig. S1D).

### *ENO2* promoted MSI-H CRC cells migration and glycolytic function

**Fig. 3:**
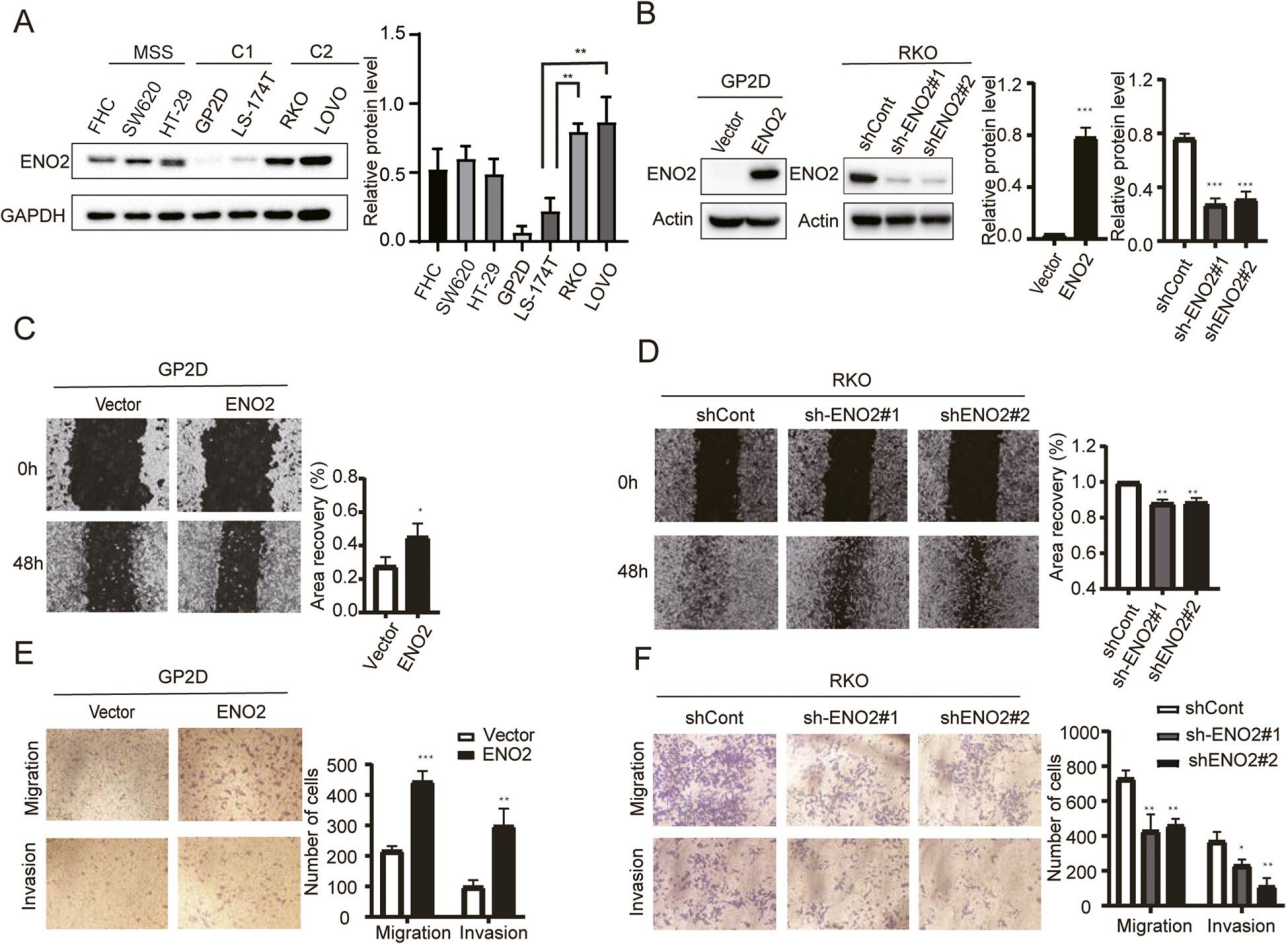
*ENO2* was involved in the migration and invasion of CRC cells. **A** Expression levels of ENO2 in six colorectal cancer cell lines (SW620, HT29, GP2D, LS-174 T, RKO,LOVO) and one normal colon cell line (FHC). **B** Transfection efficiency of lentiviral vectors in GP2D and RKO cells was validated by western blotting. **C** and **D** Migration of GP2D and RKO cells was measured by wound healing assay. **E** and **F** Invasion and migration of GP2D and RKO cells were measured by transwell assays. Data are shown as mean ± SD; *p < 0.05, **p < 0.01, and ***p < 0.001

To explore the the relationship between ENO2 and MS status, We analyzed the expression levels of ENO2 in patients with MSI and MSS. The results showed that ENO2 was more expressed in patients with MSI-H CRC(Fig. S2A). Besides, the endogenous expression of ENO2 protein in different cell lines was examined. The results showed that the expression of ENO2 in MSS CRC cell lines was low and uniform, while MSI-H CRC cell lines were quite different, with low ENO2 expression in the C1 subtype cell lines and high ENO2 expression in the C2 subtype cell lines (Fig. 3A). In summary, there is a certain correlation between ENO2 expression and MSI-H, but the relationship between function and MS status is unclear. Subsequently, the *ENO2* expression was upregulated in GP2D cells and knocked down in RKO cells via lentiviral vectors (Fig. 3B). Wound healing and transwell assays demonstrated that *ENO2* overexpression significantly enhanced cell migration and invasion. Conversely, knocking down *ENO2* in RKO cells resulted in a significant inhibition of cell migration and invasion (Fig. 3C-F). Therefore, these findings strongly suggest that *ENO2* plays a crucial role in regulating the migration and invasion processes in MSI-H CRC.

To further define the effect of ENO2 on aerobic glycolysis in MSI-H CRC cells, measurements of the extracellular acidification rate (ECAR) were performed. ENO2 overexpressing cells exhibited an enhanced glycolysis phenotype with obvious increases in glycolytic capacity (Fig. S3A). Glycolytic enzymes, including hexokinase (HK1/2), glucose-6-phosphate isomerase (G6PI), pyruvate dehydrogenase kinase 1 (PDK1) and lactate dehydrogenase (LDHA/LDHB), are critical regulators of the glycolytic pathway. We next investigated the expression profiles of these genes in ENO2 overexpressing cells. Interestingly, overexpression of ENO2 increased the HK2 and PDK1 protein levels (Fig. S3B). These findings indicated that ENO2 might enhances MSI-H CRC cell glycolysis by regulating glycolytic enzymes.

### *ENO2* regulated EMT via the PI3K-AKT signaling pathway

**Fig. 4:**
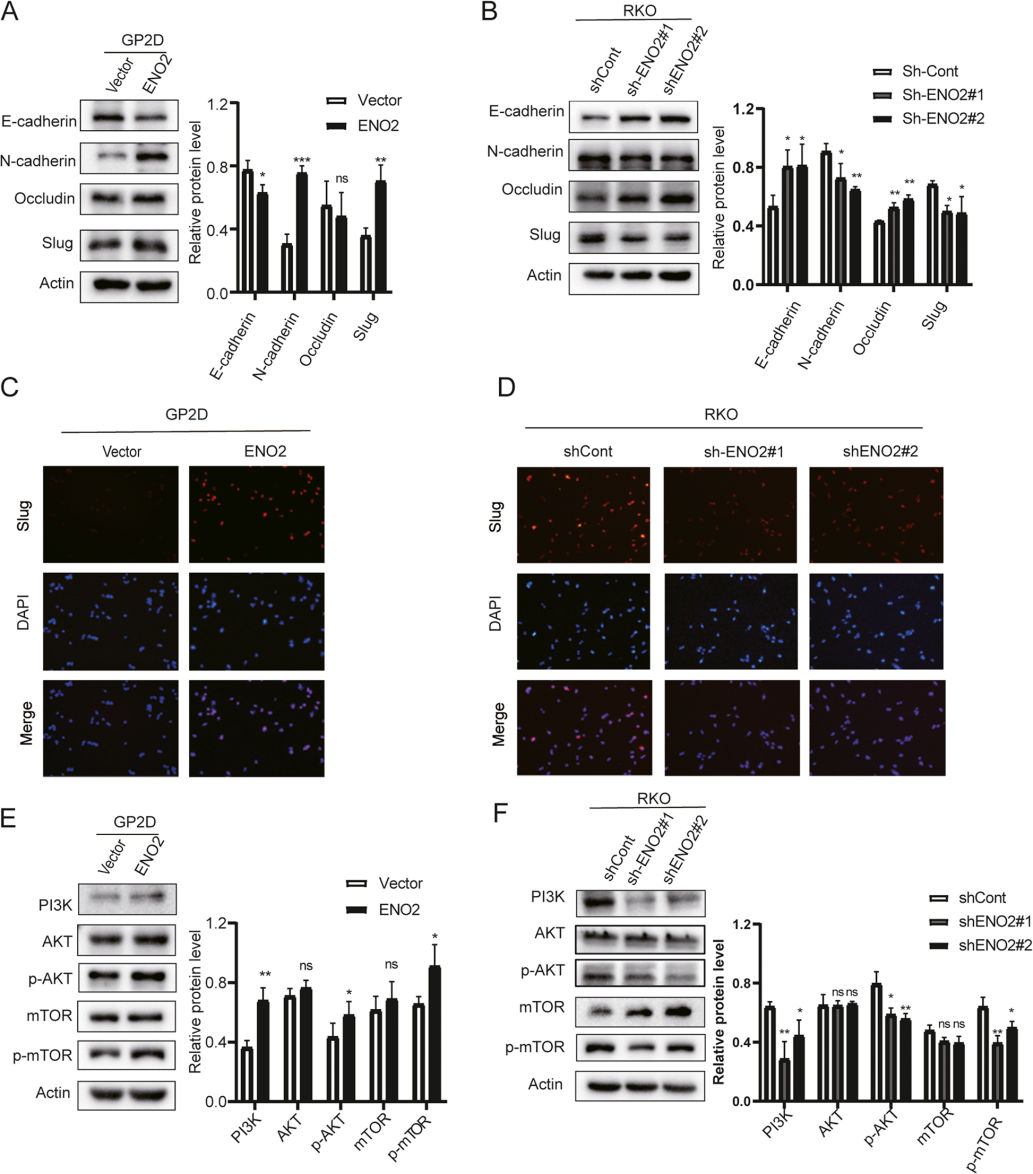
The effects of *ENO2* on EMT are partially dependent upon the PI3K/AKT signaling pathway in tumor cells. **A** and **B** Western blots showing the levels of EMT-associated proteins, *E-cadherin*, *N-cadherin*, *Occludin*, and *Slug*. **C** and **D** Protein expression and sub-localization of *Slug* as visualized by immunofluorescence. **E** and **F** Levels of *PI3K*, *AKT*, *p-AKT*, *mTOR,* and *p-mTOR* proteins were assessed in stably-transfected GP2D and cells. Data are shown as mean ± SD; ns: no statistically significant difference; *p < 0.05, **p < 0.01, and ***p < 0.001

EMT is a critical process in which epithelial cells acquire a mesenchymal phenotype, occurring in specific physiological and pathological contexts. In colorectal cancer (CRC), EMT is recognized as a pivotal molecular mechanism contributing to metastasis [22]. Thus, we investigated the expression levels of EMT-associated molecules. Our findings confirmed that the overexpression of ENO2 in CRC cells resulted in decreased expression of E-cadherin and increased expression of N-cadherin and SLUG (Fig. 4A, C). Conversely, ENO2 knockdown in RKO cells led to elevated expression of epithelial markers and decreased expression of mesenchymal markers (Fig. 4B, D).

Given the PI3K-AKT signaling pathway activity of the C2 subtype is upregulated (Fig. S4), and considering that the PI3K-AKT pathway is known to promote tumor metastasis by regulating EMT [23], we further investigated this relationship. Our results demonstrated that ENO2 overexpression increased the expression levels of PI3K, p-AKT, and p-mTOR (Fig. 4E), while ENO2 knockdown had the opposite effect, inhibiting the expression of these proteins (Fig. 4F). Overall, these findings suggest that ENO2-induced EMT is partially dependent on the PI3K/AKT/mTOR signaling pathway.

### ENO2 correlated with clinicopathological features

**Fig. 5:**
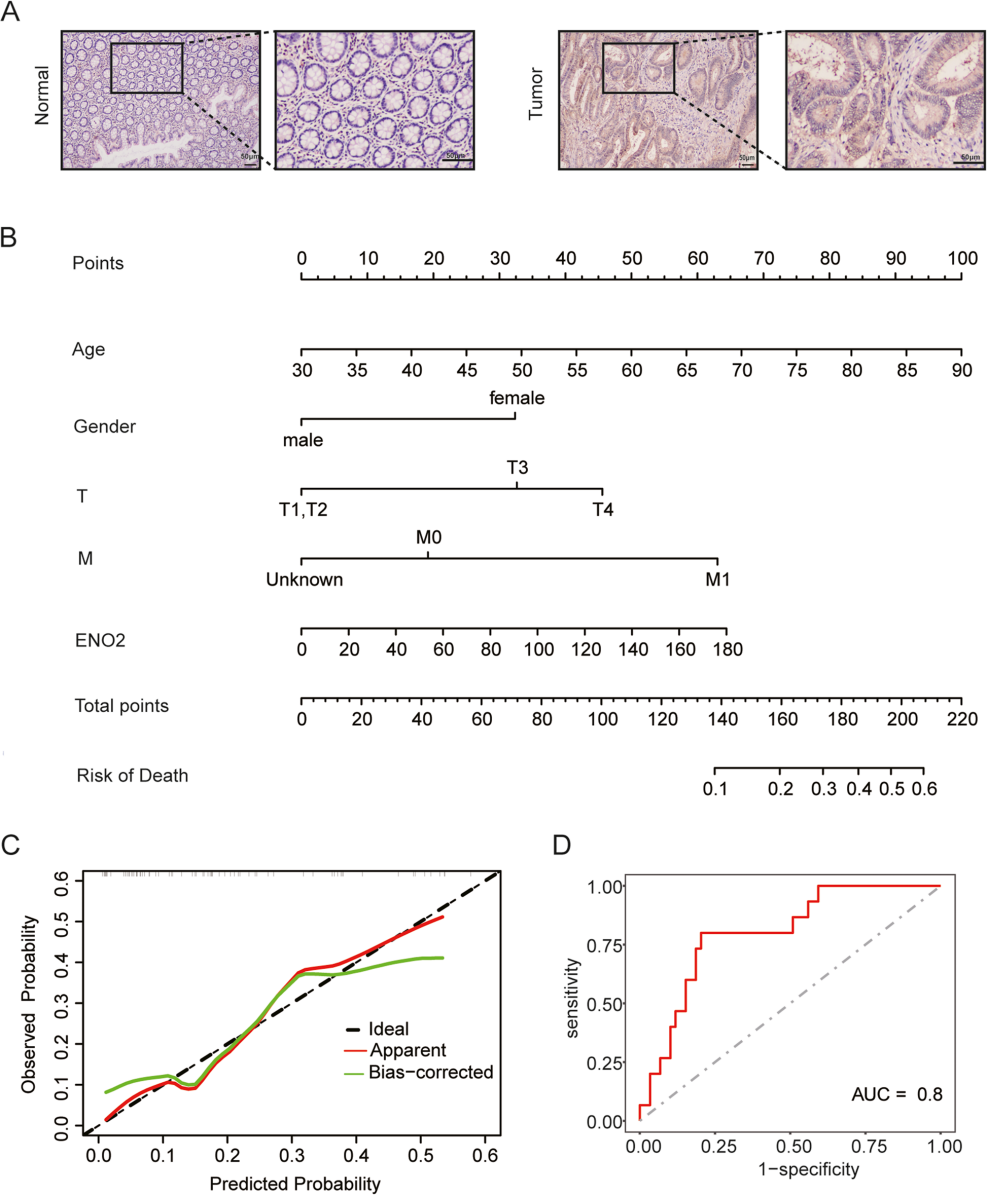
The assessment and validation of *ENO2* as a prognostic factor. **A** Immuno-histochemical staining of *ENO2* protein in MSI-H CRC tissue and adjacent normal tissue (Scale bar, 50 μm). **B** Nomogram for predicting the death of MSI-H CRC patients. **C** Calibration plot of the nomogram. **D** The ROC curve to predict the death of MSI-H CRC patients based on the nomogram

The results of IHC staining demonstrated ENO2 protein expression in tumor tissues is higher than that in adjacent normal tissues (Fig. 5A). The association between ENO2 expression and clinical features was then explored, revealing a remarkable correlation between ENO2 expression and T stage and perineural invasion at the protein level (Table 1).

**Table 1:**
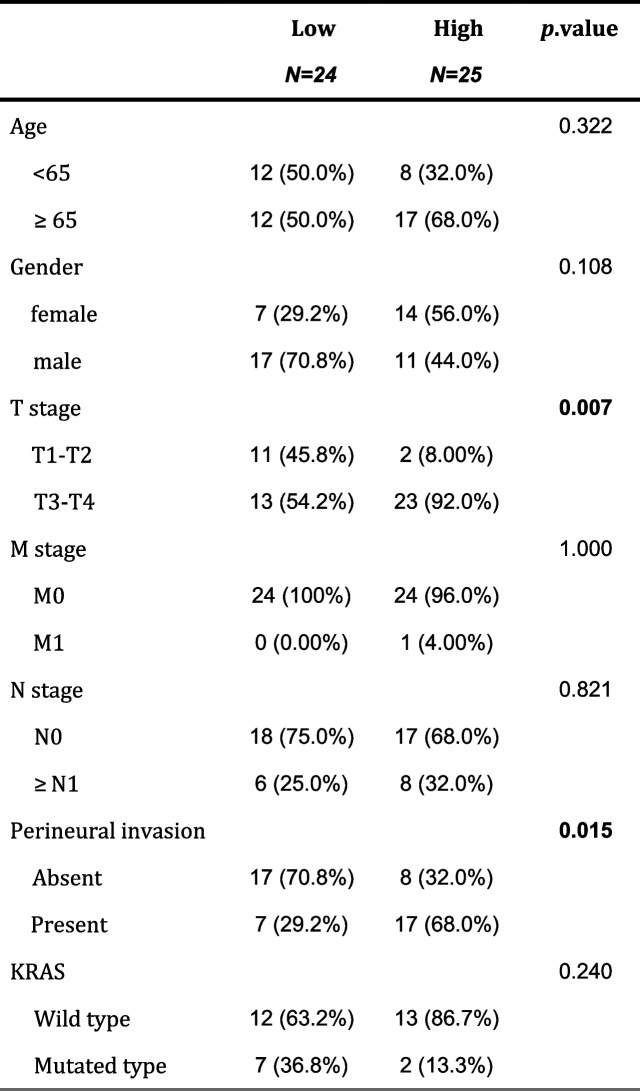
The association between ENO2 protein level and clinicopathological features of MSI-H CRC patients (*n* = 49)

### Evaluation of *ENO2* as a prognostic factor in MSI-H CRC patients

A nomogram was developed using the TCGA dataset, incorporating the expression of ENO2 and clinical characteristics such as age, gender, tumor (T) and metastasis (M) stage. The total points obtained from scoring these features were inversely correlated with the observed survival probability, indicating that higher total points were associated with poorer survival (Fig. 5B). The nomogram's prognostic efficacy was validated by calibration curves and ROC curve (AUC = 0.8), demonstrating a favorable prognostic effect (Fig. 5C and D).

## Discussion

Some MSI-H CRC patients do not gain overall survival benefits from immune checkpoint-blockade treatment. In other words, the heterogeneity of MSI-H CRC is still limited for prognostic assessment [24]. Screening novel biomarkers to help estimate prognosis remains critical and urgent. In this study, we conducted a comprehensive analysis of scRNA-seq and bulk RNA-seq to discover a biomarker that exhibited excellent prognostic value in MSI-H CRC.

In this study, MSI-H CRC cell lines were divided into distinct subtypes by UMAP based on the metabolism-related gene expression profile. Compared with C1 subtype, C2 had multiple upregulated metabolic pathways, including metabolism of carbohydrates, integration of energy metabolism, etc. Previous studies have revealed that oncogenic signals driving CRC progression have been implicated in the control of specific metabolic pathways in CRC and other tumor types [25, 26]. Therefore, the exploration of metabolism-related subtypes may reveal metabolic heterogeneity, which may help to explain the survival heterogeneity among MSI-H CRC patients. Further analysis revealed that C2 showed up-regulated activity of the EMT pathway, which has been confirmed to be associated with CRC metastasis [20]. Survival analysis showed that patients with C2 subtype had shorter overall survival and poorer prognosis than C1 subtype, suggesting that this subtype may be highly malignant. To identify metabolic markers of the C2 subtype, the DEGs between the C1 and C2 subtypes were identified, and *ENO2* was identified as a marker gene for the C2 subtype.

ENO2 is a metal-activated metalloenzyme that catalyzes the dehydration of 2-phosphate-D-glycerate (PGA) to phosphoenolpyruvate (PEP) in the glycolytic pathway and is implicated in the ondrogenesis of several malignancies [27]. Many studies proved that *ENO2* was essential in cancer progression. For example, *ENO2* was involved in the oncogenic process of BRAF V600E colorectal cancer by regulating the MAPK/ERK signaling pathway and may be a novel therapeutic target for BRAF V600E mutant colorectal cancer [28]. Moreover, ENO2-derived phosphoenolpyruvate functions as an endogenous inhibitor of HDAC1 and confers resistance to antiangiogenic therapy [29]. Although the role of ENO2 in metastasis and chemotherapy resistance has been well-studied, how ENO2 takes part in glucose mechanism remains unknown. In this study, we demonstrated that ENO2 might enhances MSI-H CRC cell glycolysis by regulating glycolytic enzymes. However, there are two limitations of this study. On the one hand, the sample size of scRNA-seq data is relatively small. On the other hand, the regulatory mechanisms of *ENO2* in MSI-H CRC remain ambiguous, and which is exactly what future work arising from this study should continue to explore.

In conclusion, two metabolism-related subtypes of MSI-H CRC were reavealed, and ENO2 was identified as the marker gene of C2 subtype. ENO2 promoted MSI-H CRC cell migration, invasion, glycolysis, and epithelial-mesenchymal transition (EMT). These findings may deepen our understanding regarding the role of ENO2 in MSI-H CRC. Besides, ENO2 has reference significance in predicting the progression in MSI-H CRC. It is promising to become a novel biomarker for the prognosis of MSI-H CRC.

### Supplementary Information

Supplementary Material 1.

Supplementary Material 2.

## Data Availability

The original contributions presented in the study are included in the article/Supplementary Material, further inquiries can be directed to the corresponding authors.

## Abbreviations

CRC: Colorectal cancer
MSI-H: Microsatellite instability-high
MSI-L: Microsatellite instability-low
MSS: Microsatellite stability
*ENO2*: Enolase 2
GEO: Gene Expression Omnibus
TCGA: The Cancer Genome Atlas
EMT: Epithelial-mesenchymal transition
ECAR: Extracellular Acidification Rate
AUC: Area under curve
DEG: Different expression gene
