# Supplementary material for: Investigation of ENO2 as a promising novel marker for the progression of colorectal cancer with microsatellite instability-high
Source: BMC Cancer. 2024 May 9;24:573. doi: 10.1186/s12885-024-12332-4 (PMC11080076; doi:10.1186/s12885-024-12332-4)
Supplement: Supplementary file 1 — Supplementary Material 1. [file 12885_2024_12332_MOESM1_ESM.pdf]

Supplementary Information

Additional file 1: Figure S1.

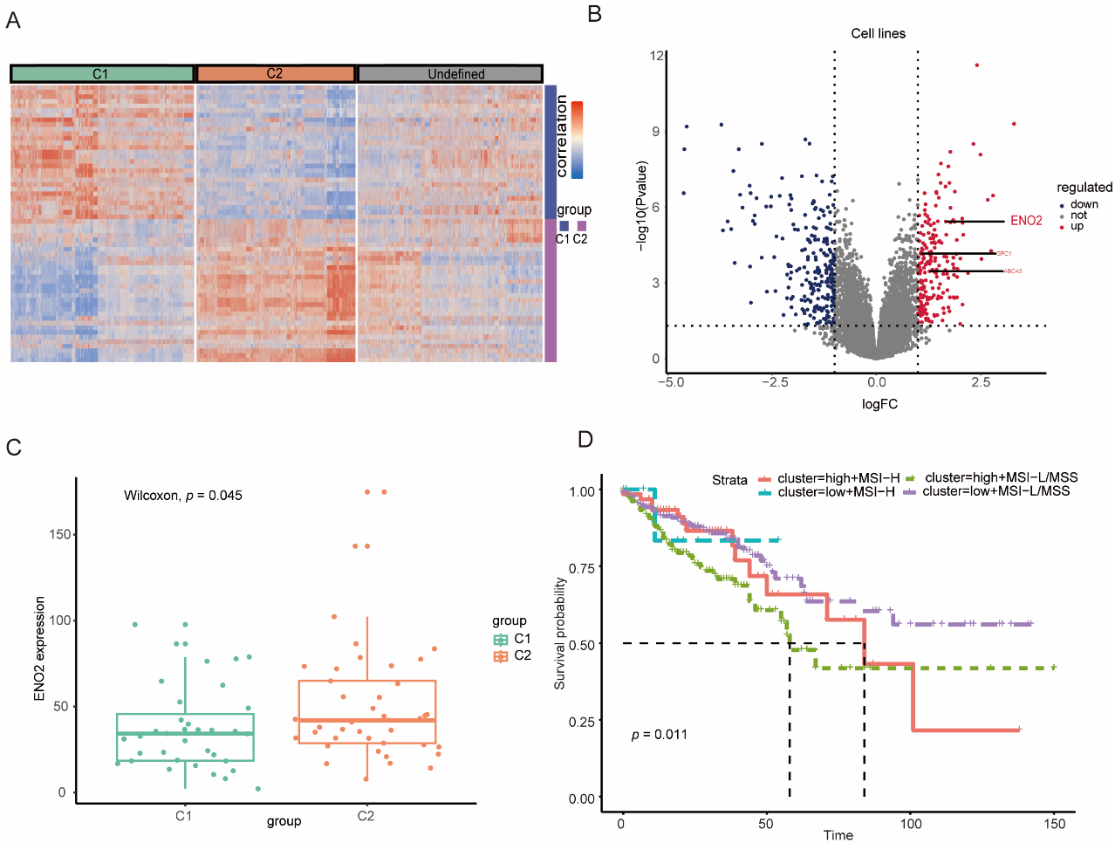

Figure S1. Subtype analysis and identification of the hub gene. (A) Heat map of correlation between CRC cell subpopulations and cell line types. (B) Differential gene analysis in bulk transcriptomics. (C) Expression of ENO2 between C1 subtype and C2 subtype. (D) Kaplan-Meier curves for patients stratified by microsatellite status and ENO2 expression.

Additional file 2: Figure S2.

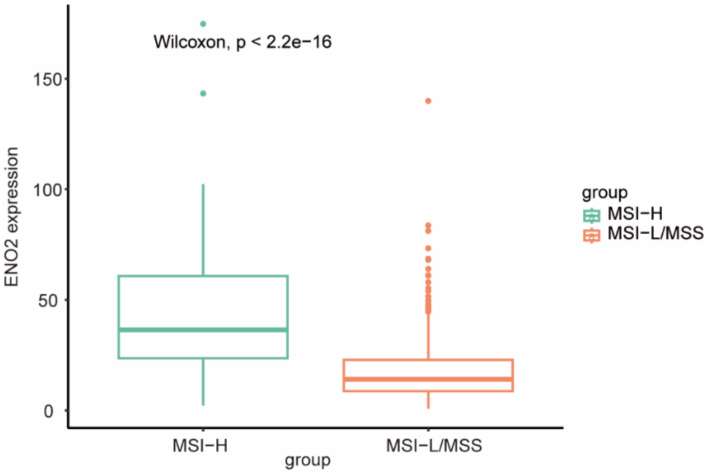

Figure S2. Expression of ENO2 in TCGA MSI-H and MSS cohort.

Additional file 3: Figure S3.

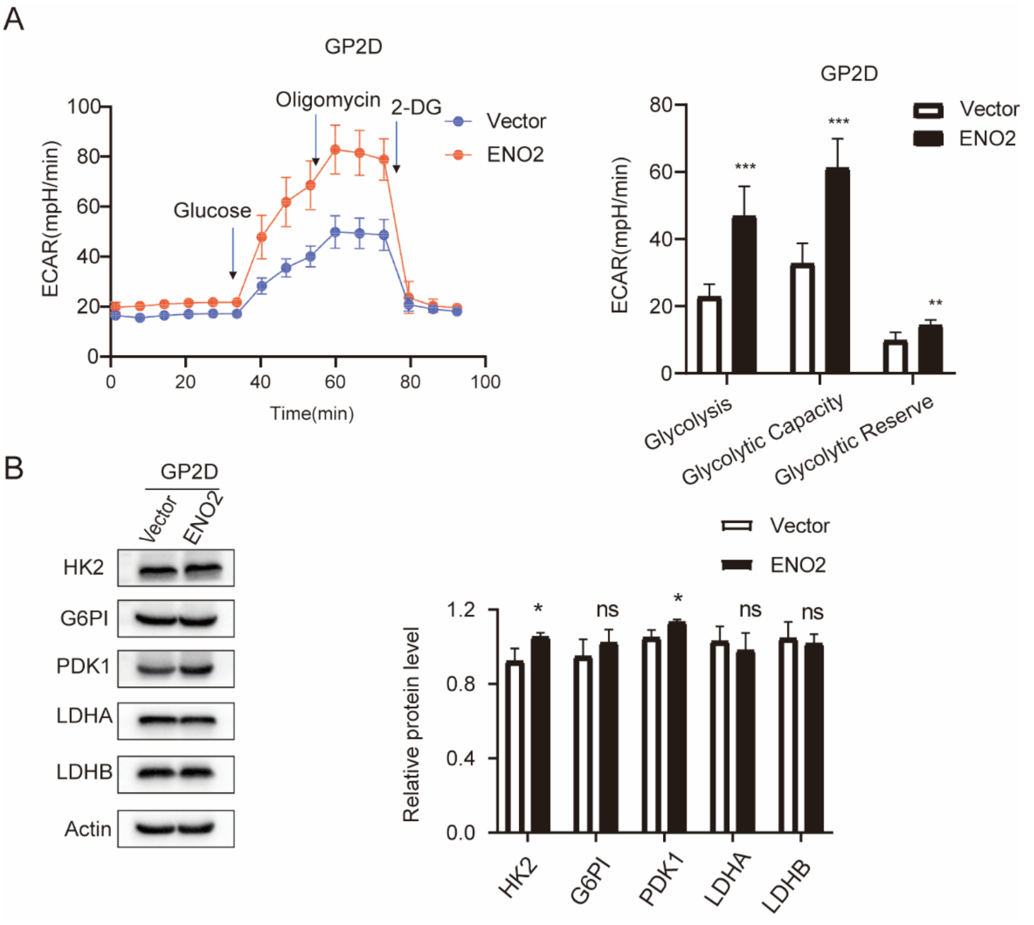

Figure S3. ENO2 enhanced MSI-H CRC cell glycolysis by regulating glycolytic enzymes. (A) Changes in ECAR levels in ENO2-overexpressing GP2D cells. (B) Western blot analysis of glycolytic enzymes protein levels in ENO2-overexpressing GP2D cells.

Additional file 4: Figure S4.

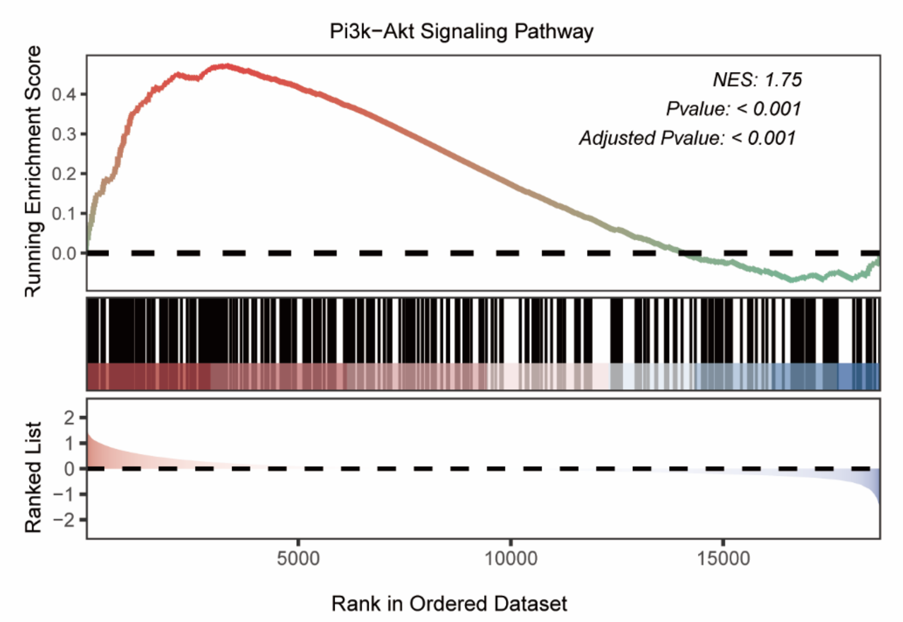

Figure S4. GSEA of PI3K-AKT.
