# Supplementary material for: Investigation of ENO2 as a promising novel marker for the progression of colorectal cancer with microsatellite instability-high
Source: BMC Cancer. 2024 May 9;24:573. doi: 10.1186/s12885-024-12332-4 (PMC11080076; doi:10.1186/s12885-024-12332-4)
Supplement: Supplementary file 2 — Supplementary Material 2. [file 12885_2024_12332_MOESM2_ESM.pdf]

Origin images of WB

Figure 3A（The results of the triplicate experiment are as follows, the images in the red box are images of the body text, and the images in the blue boxes are duplicates.）

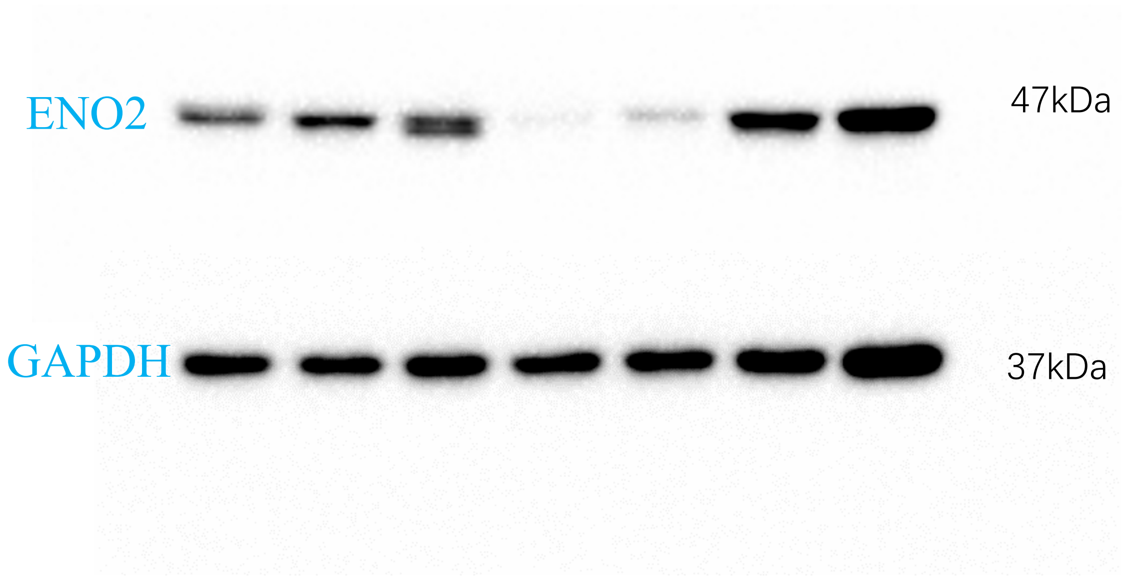

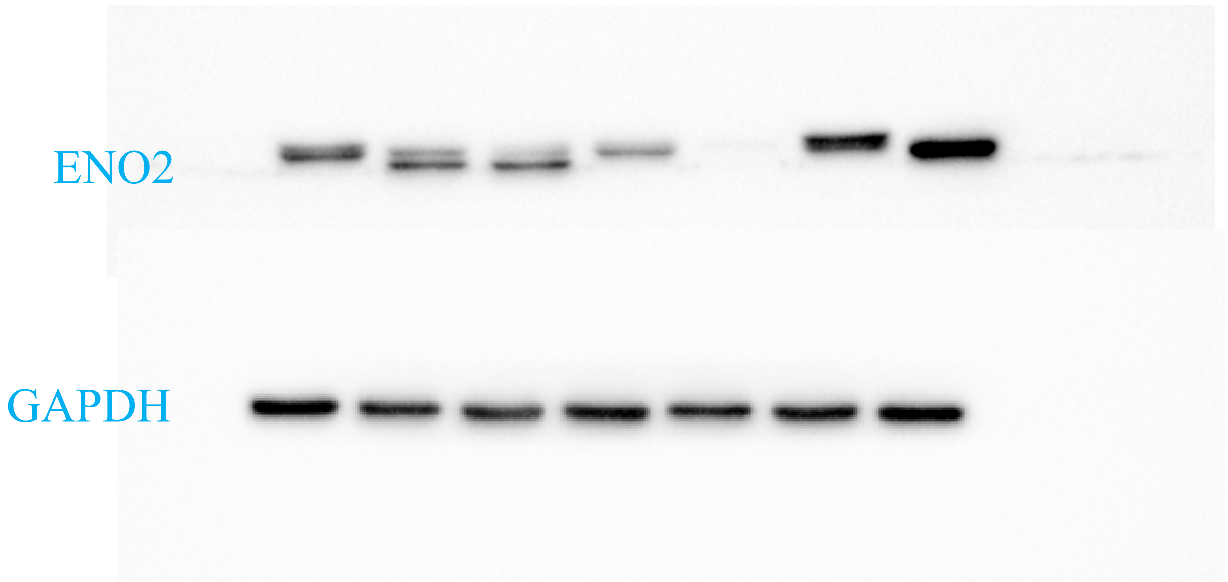

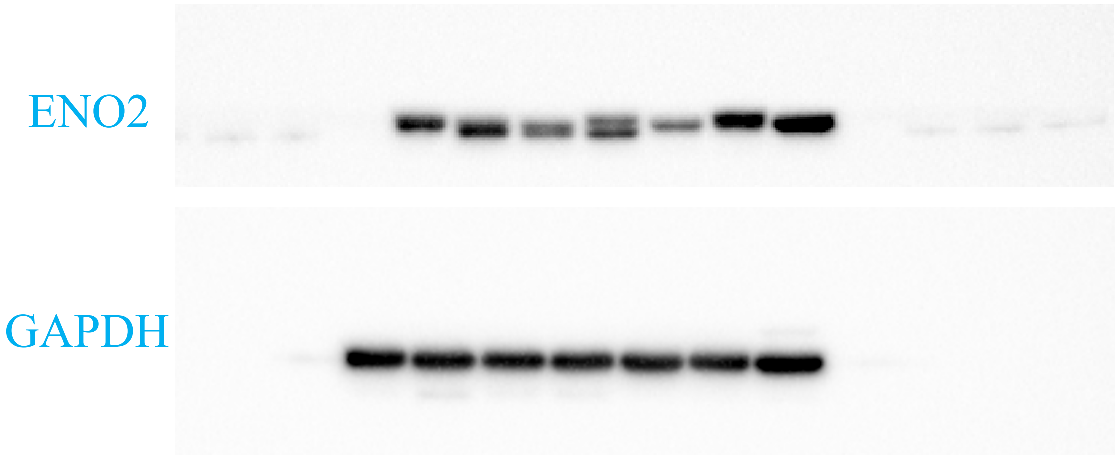

Figure 3B

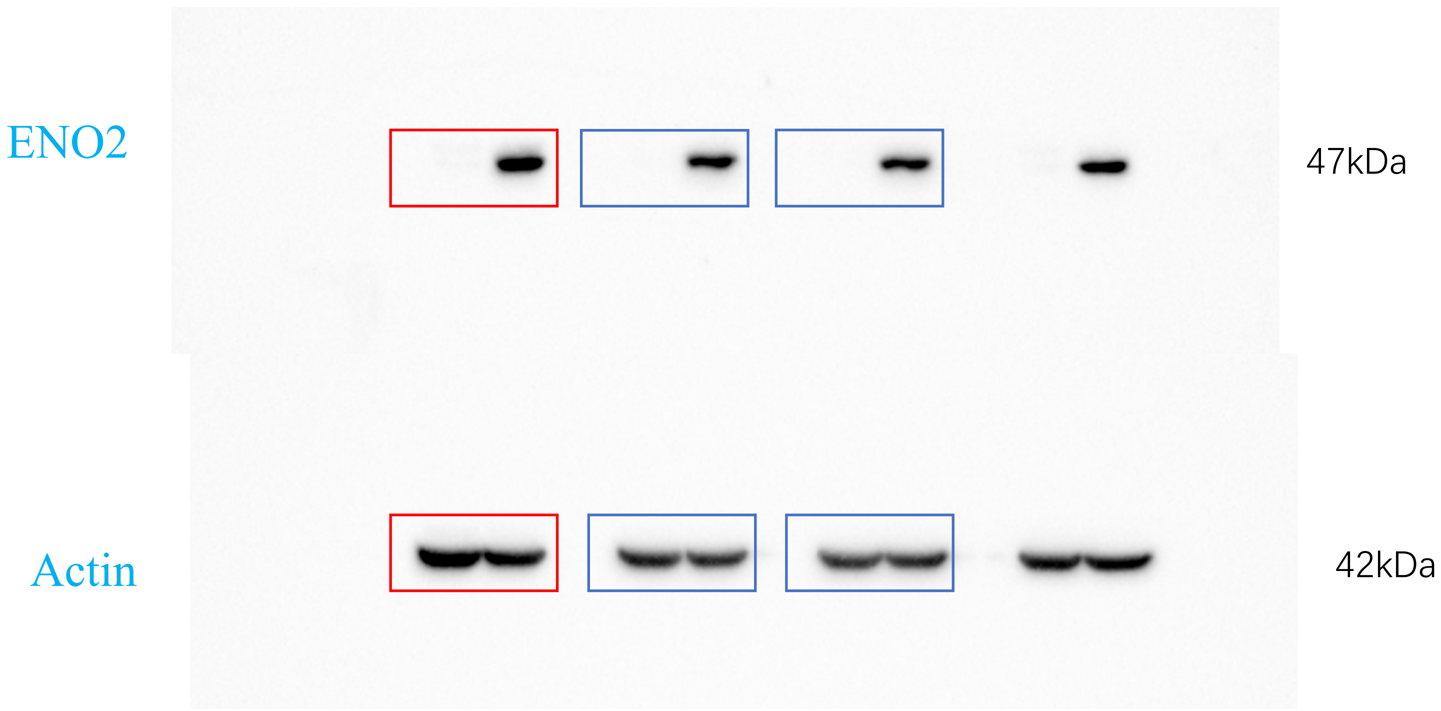

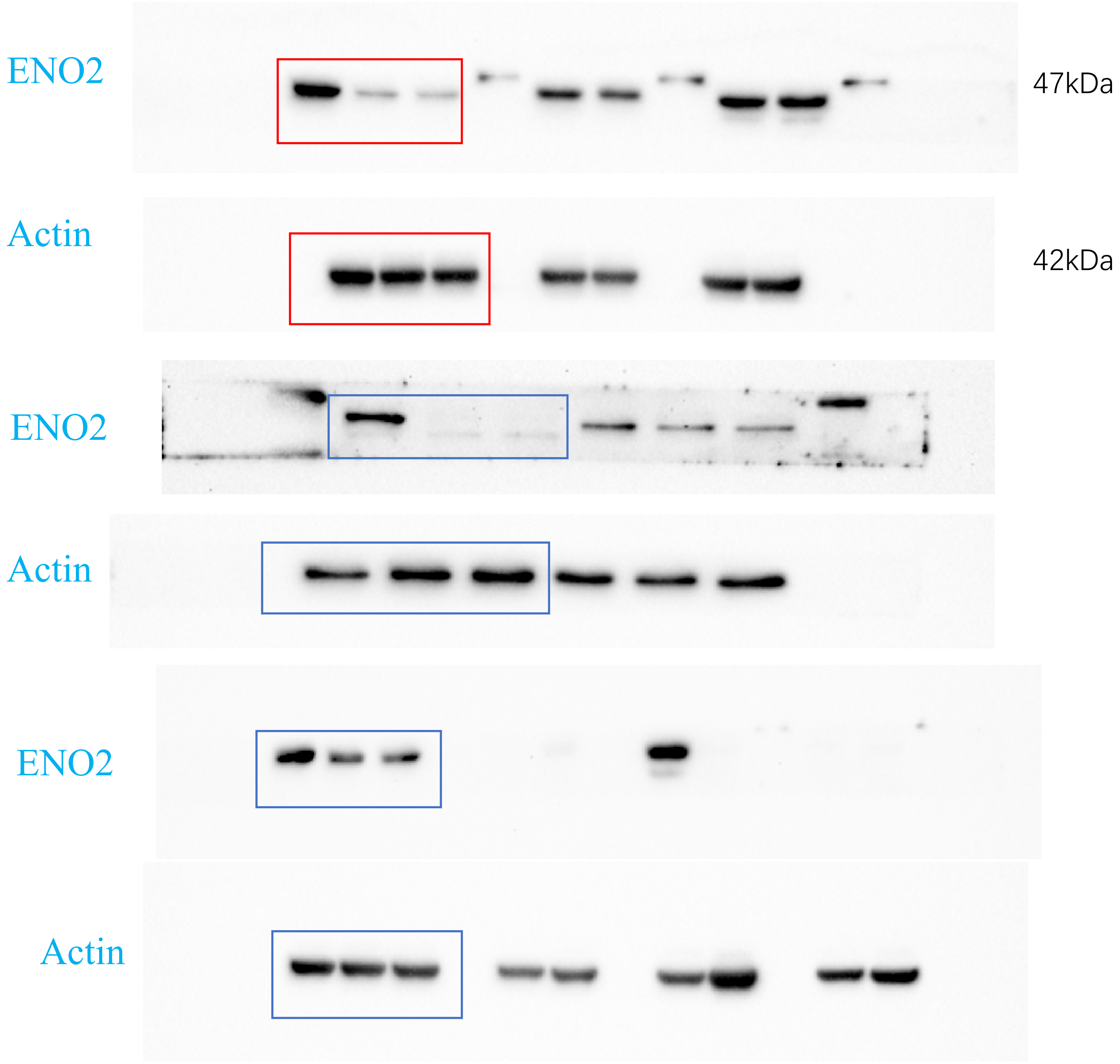
ENO2
47kDa
Actin
42kDa
ENO2
Actin
ENO2
Actin

Figure 4A

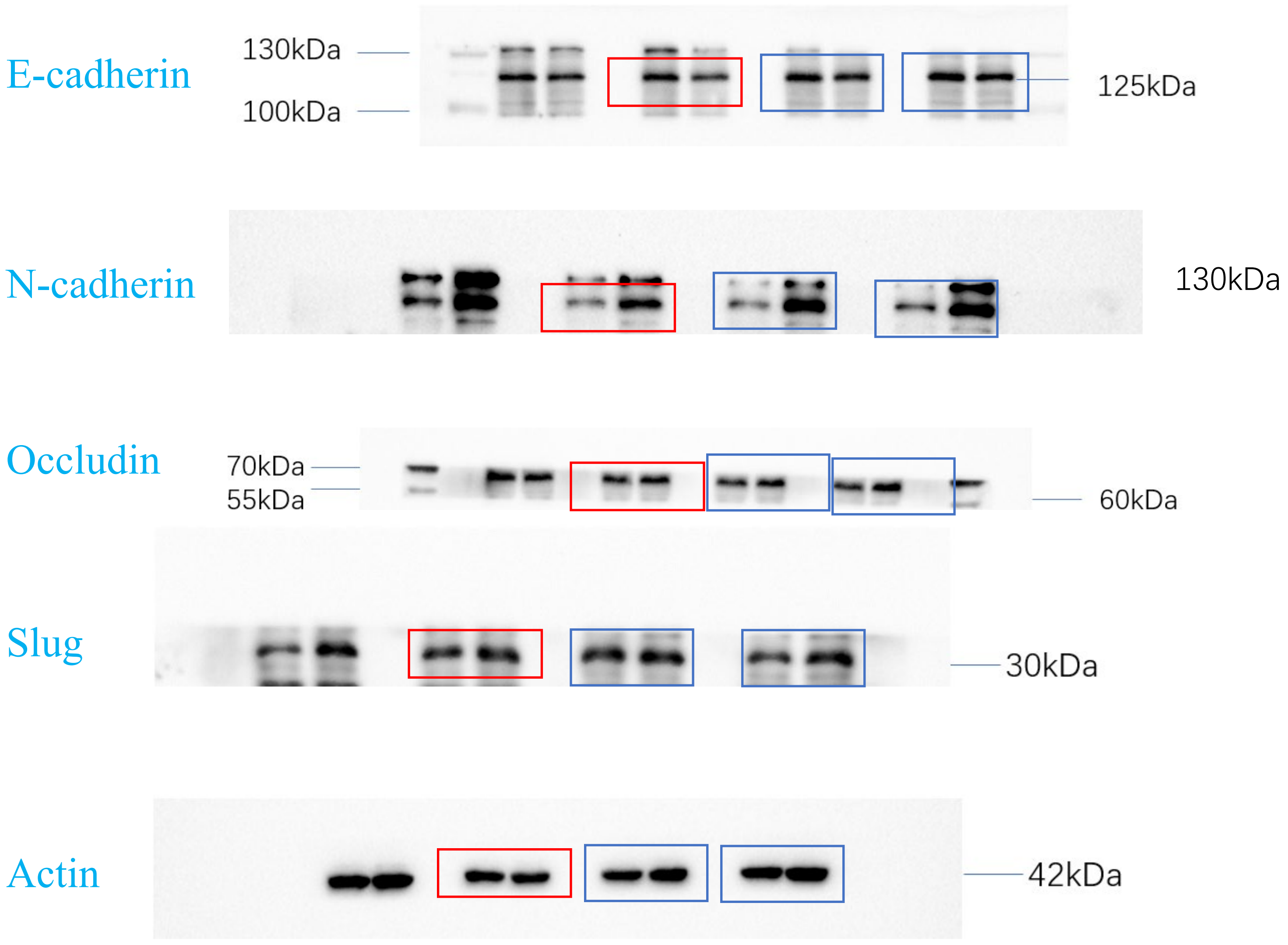

E-cadherin
130kDa
100kDa
125kDa
N-cadherin
130kDa
Occludin
70kDa
55kDa
60kDa
Slug
30kDa
Actin
42kDa

Figure 4B

E-cadherin

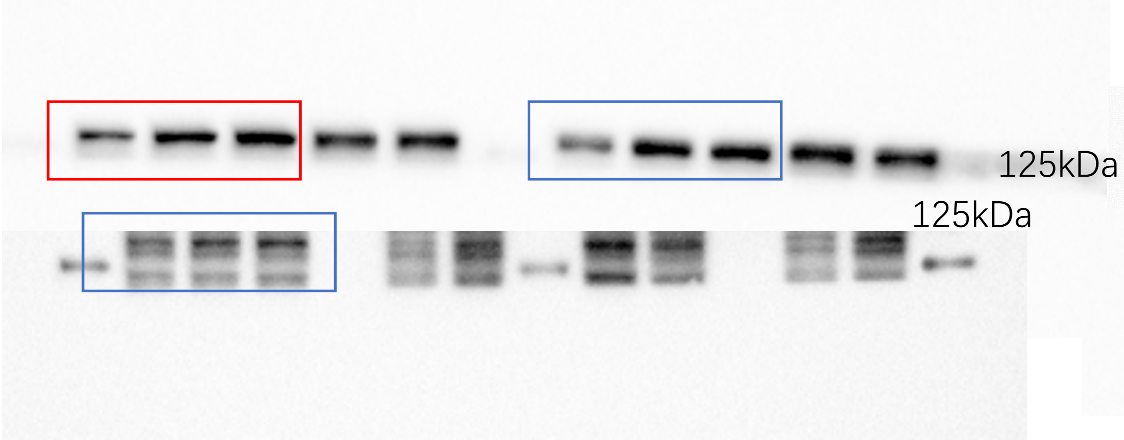

Actin corresponding to E-CA

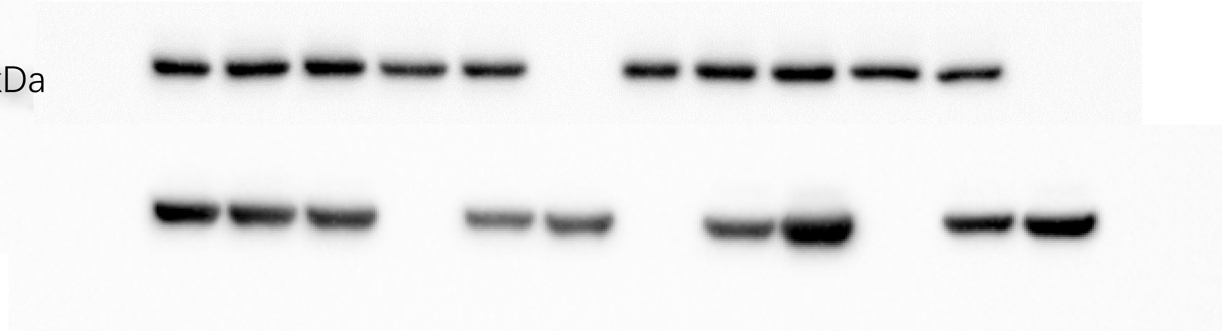

N-cadherin

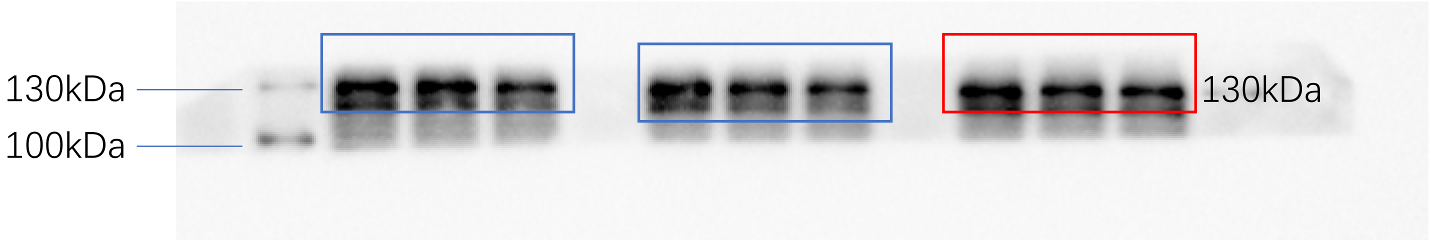

Occludin

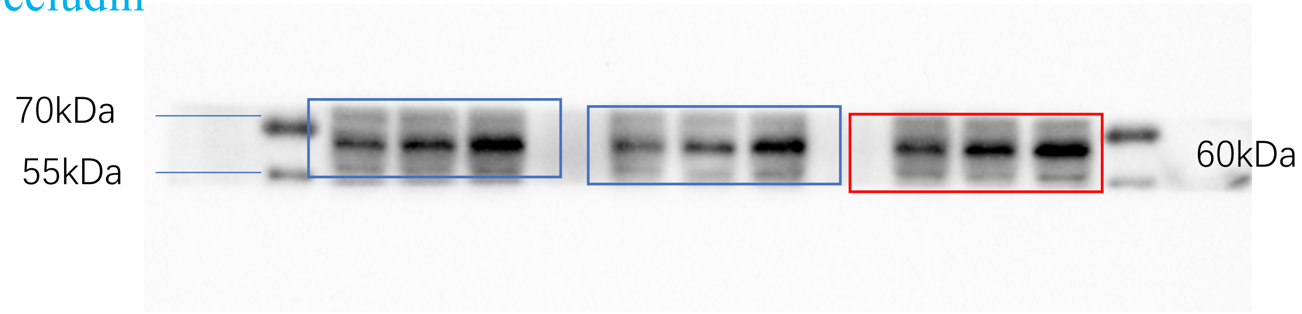

Slug

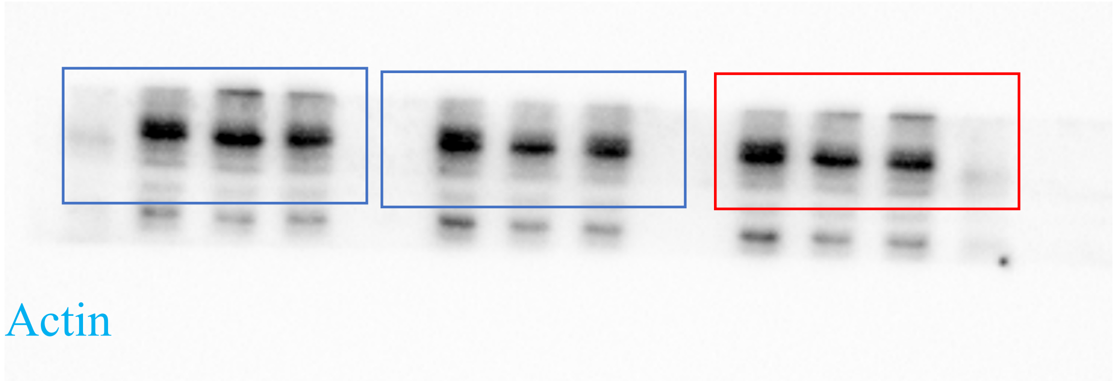

30kDa

Actin

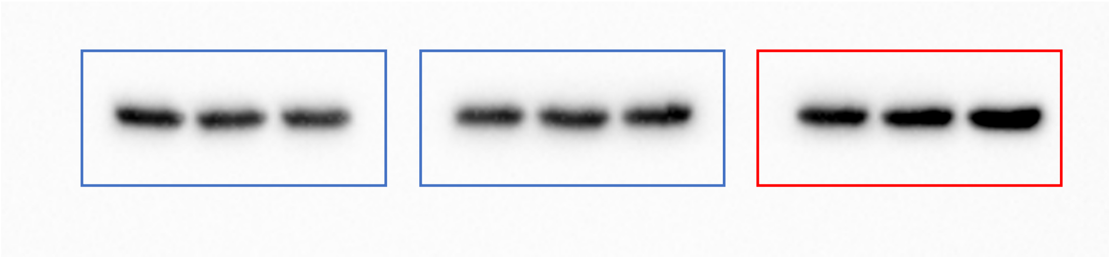

42kDa

Figure 4E

PI3K

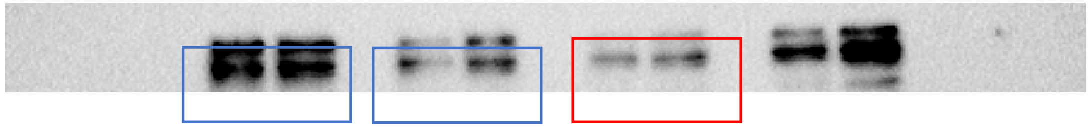

110kDa

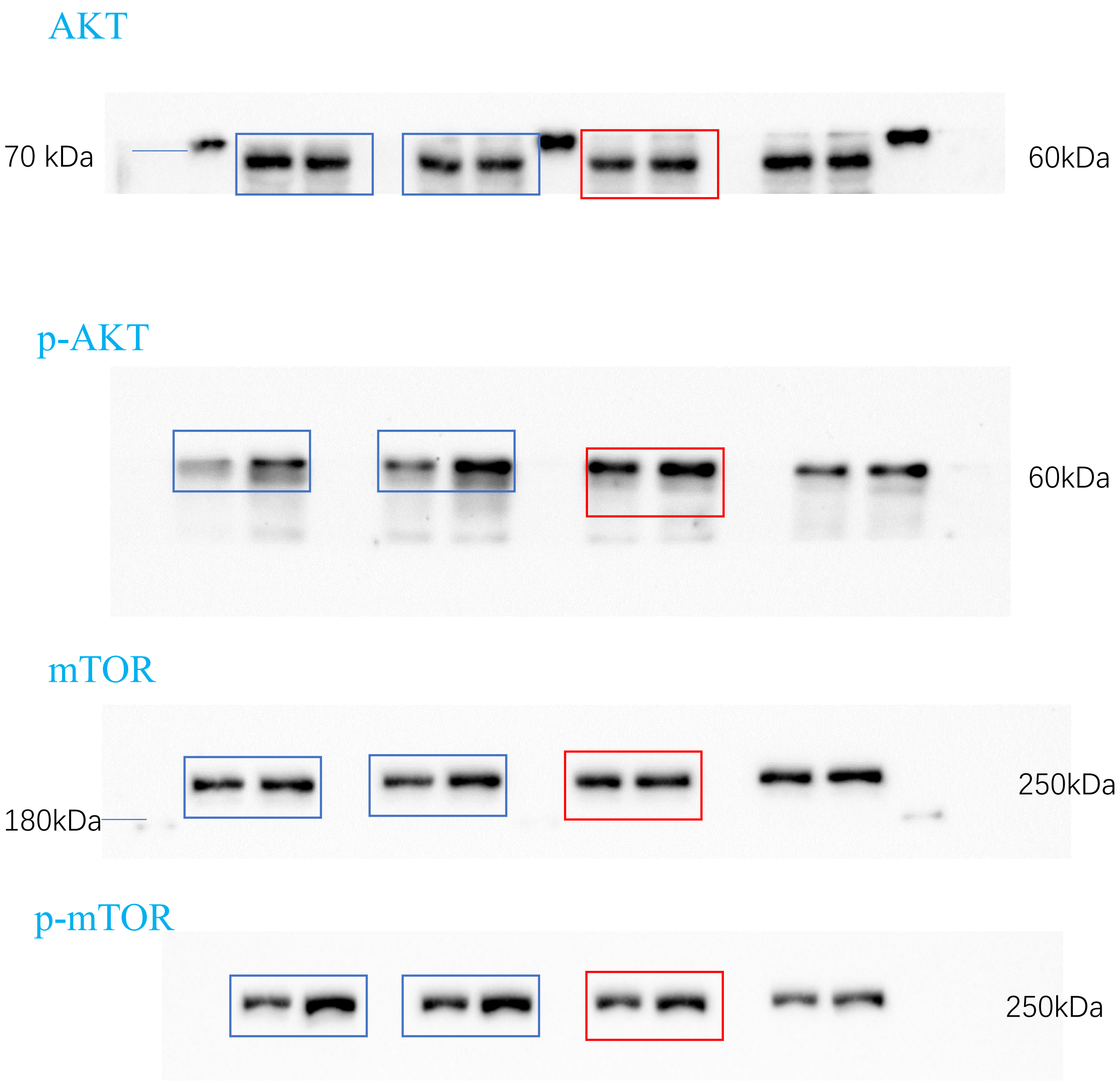
AKT
70 kDa
60kDa
p-AKT
60kDa
mTOR
250kDa
180kDa
p-mTOR
250kDa

Actin

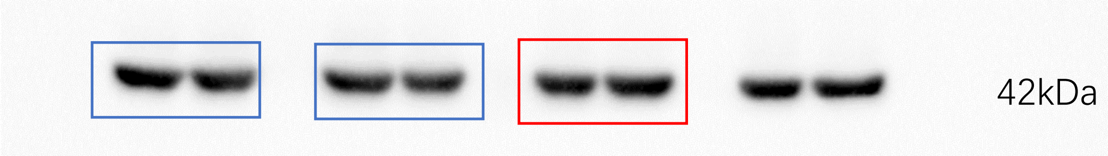

Figure 4F

PI3K

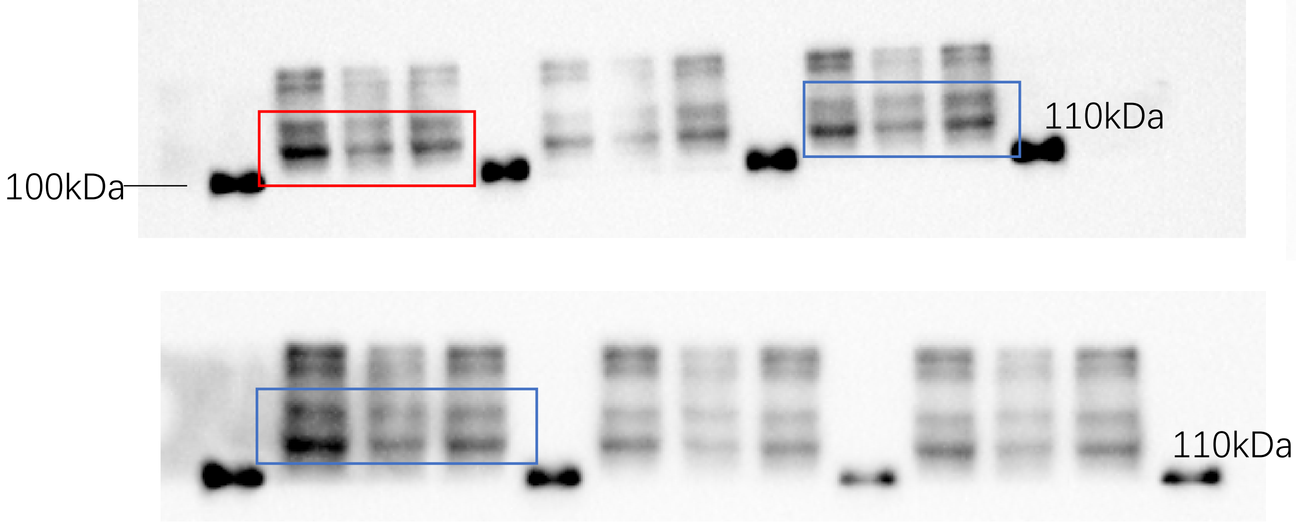

Actin corresponding to E-CA

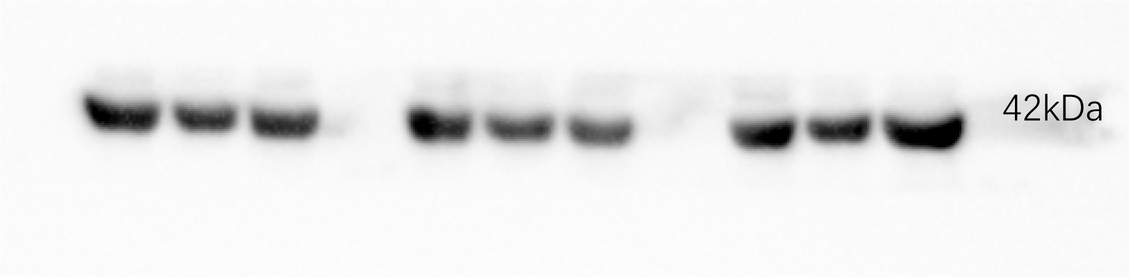

AKT

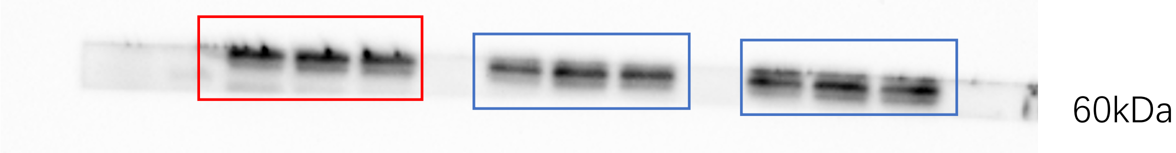

p-AKT

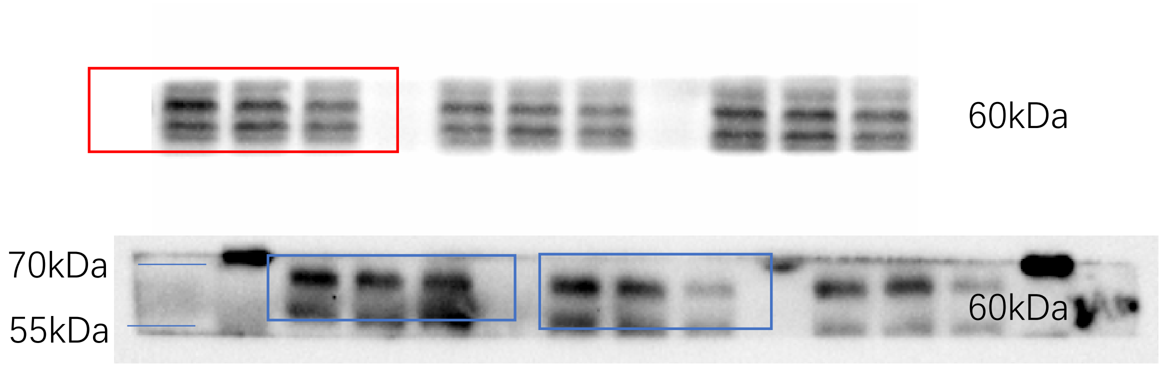

Actin corresponding to E-CA

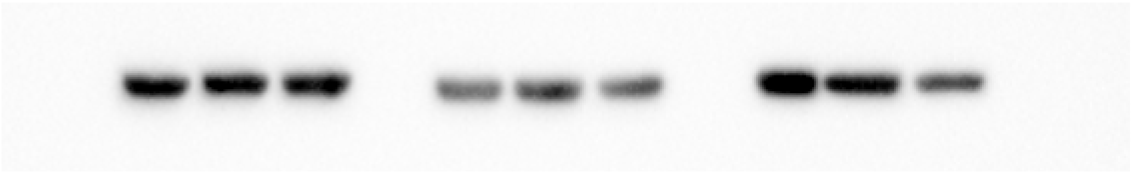

mTOR

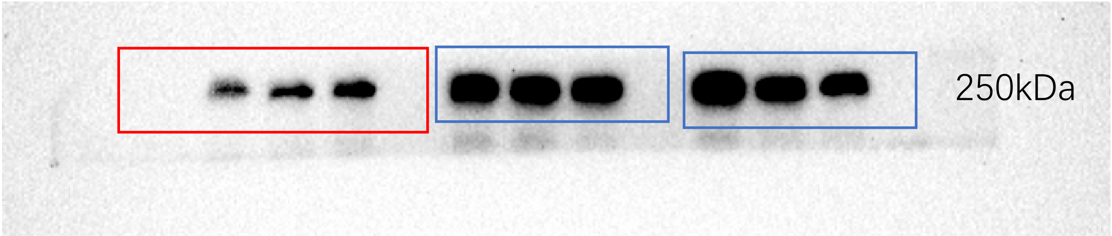

p-mTOR

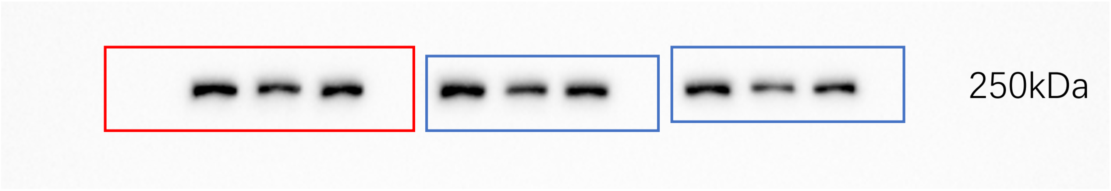

Actin

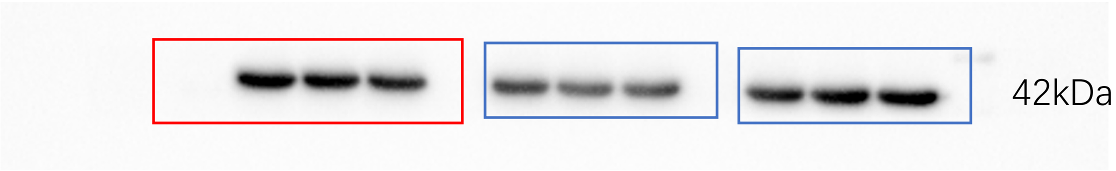

Figure S3 B

HK2

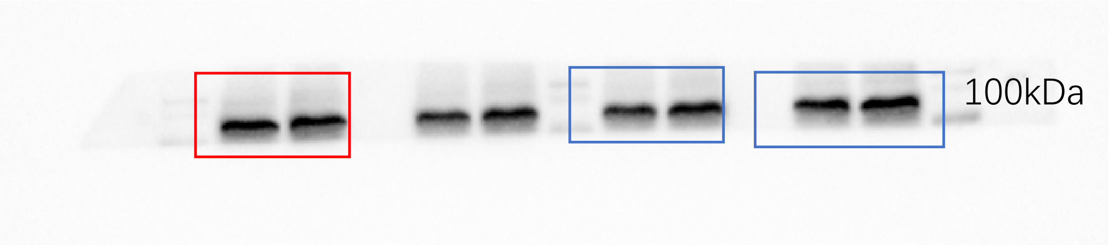

G6PI

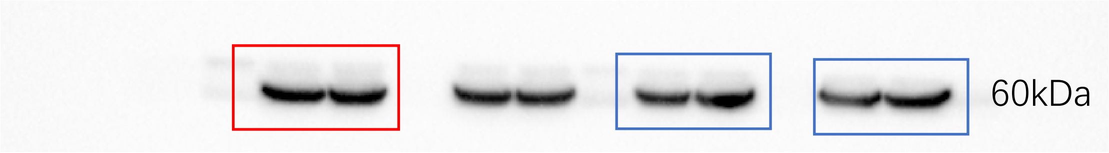

PDK1

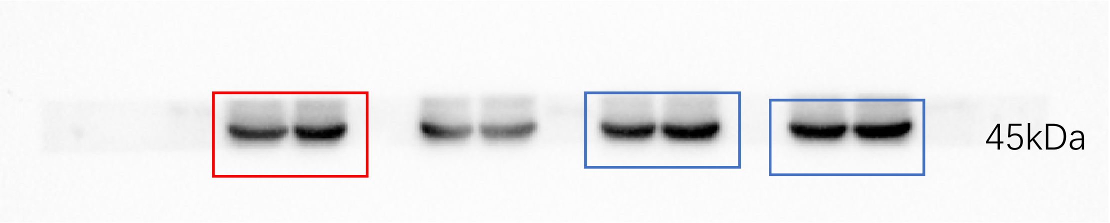

LDHA

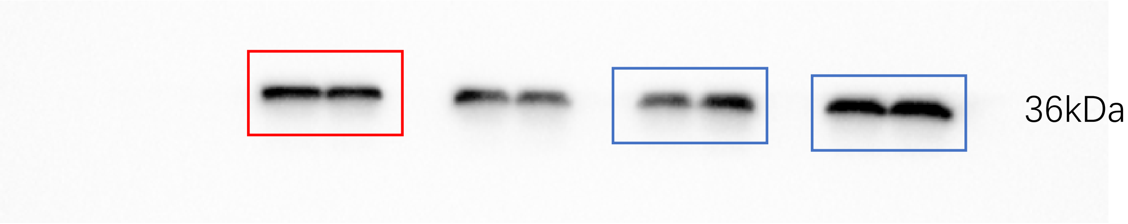

LDHB

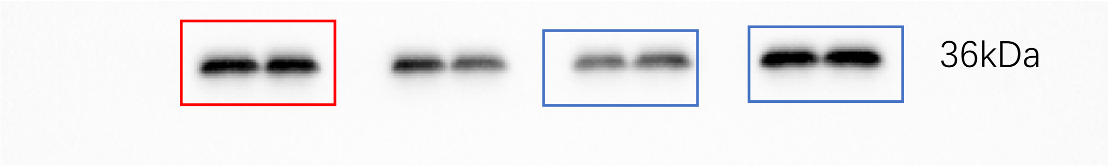

Actin

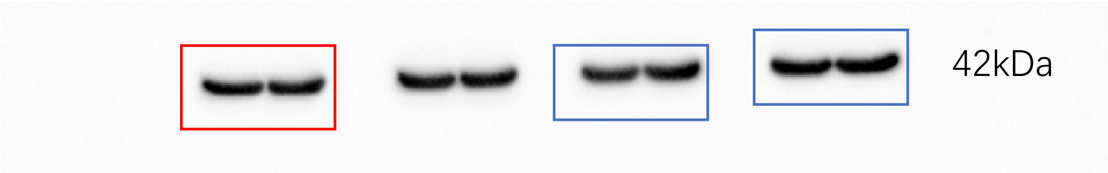

The primary antibodies used for Western blot analysis were as follows: ENO2 (66150-1-Ig, 1:1000), Beta-Actin (20536-1-AP, 1:5000), E-cadherin (20874-1-AP, 1:5000), N-cadherin (22018-1-AP, 1:3000), Occludin (27260-1-AP, 1:1000), Slug (12129-1-AP, 1:2000), PI3K (67071-1-Ig, 1:1000), AKT (10176-2-AP, 1:2000), p-AKT Ser473(66444-1-Ig, 1:1000), mTOR (66888-1-Ig, 1:1000), p-mTOR Ser2448(67778-1-Ig, 1:1000), HK2(22029-1-AP, 1:5000), G6PI(15171-1-AP,1:2000), PDK1(18262-1-AP, 1:1000), LDHA(21799-1-AP, 1:5000) and LDHB(14824-1-AP, 1:5000) obtained from Proteintecch.
